# Neural mechanisms and personality correlates of the sunk cost effect

**DOI:** 10.1038/srep33171

**Published:** 2016-09-09

**Authors:** Junya Fujino, Shinsuke Fujimoto, Fumitoshi Kodaka, Colin F. Camerer, Ryosaku Kawada, Kosuke Tsurumi, Shisei Tei, Masanori Isobe, Jun Miyata, Genichi Sugihara, Makiko Yamada, Hidenao Fukuyama, Toshiya Murai, Hidehiko Takahashi

**Affiliations:** 1 Department of Psychiatry, Kyoto University Graduate School of Medicine, 54 Shogoin-Kawaracho, Sakyo-ku, Kyoto 606-8507, Japan; 2 Medical Institute of Developmental Disabilities Research, Showa University, 6-11-11 Kitakarasuyama, Setagaya-ku, Tokyo, 157-8577, Japan; 3 Department of Psychiatry, The Jikei University School of Medicine, 3-25-8 Nishishinbashi, Minato, Tokyo 105-0003, Japan; 4 Division of Humanities and Social Sciences, California Institute of Technology, 1200 East California Boulevard, Pasadena, California, 91125, USA; 5 Institute of Applied Brain Sciences, Waseda University, 2-579-15 Mikajima, Tokorozawa, Saitama 359-1192, Japan; 6 School of Human and Social Sciences, Tokyo International University, 2509 Matoba, Kawagoe, Saitama 350-1198, Japan; 7 Molecular Neuroimaging Program, Molecular Imaging Center, National Institute of Radiological Sciences, 4-9-1 Anagawa, Inage-ku, Chiba 263-8555, Japan; 8 Human Brain Research Center, Kyoto University Graduate School of Medicine, 54 Shogoin-Kawaracho, Sakyo-ku, Kyoto 606-8507, Japan

## Abstract

The sunk cost effect, an interesting and well-known maladaptive behavior, is pervasive in real life, and thus has been studied in various disciplines, including economics, psychology, organizational behavior, politics, and biology. However, the neural mechanisms underlying the sunk cost effect have not been clearly established, nor have their association with differences in individual susceptibility to the effect. Using functional magnetic resonance imaging, we investigated neural responses induced by sunk costs along with measures of core human personality. We found that individuals who tend to adhere to social rules and regulations (who are high in measured agreeableness and conscientiousness) are more susceptible to the sunk cost effect. Furthermore, this behavioral observation was strongly mediated by insula activity during sunk cost decision-making. Tight coupling between the insula and lateral prefrontal cortex was also observed during decision-making under sunk costs. Our findings reveal how individual differences can affect decision-making under sunk costs, thereby contributing to a better understanding of the psychological and neural mechanisms of the sunk cost effect.

“Waste not, want not” is a rule that humans are repeatedly taught from childhood. Undoubtedly, avoiding waste is essential for allocating scarce resources efficiently. However, an overgeneralization of this highly adaptive rule can ironically result in “throwing good money after bad,” a maladaptive economic behavior known as the sunk cost effect^1^,^2^,^3^. The sunk cost effect has long been documented in various disciplines, including economics, psychology, politics, organizational behavior, and biology.

The sunk cost effect is the tendency to continue an investment, or take an action, even though it has higher future costs than benefits, if costs of time, money, or effort were previously incurred^1^. A clear example of the sunk cost effect was proposed by Arkes and Blumer (1985): Suppose that “*you have spent $100 on a ticket for a trip to Michigan. Several weeks later you buy a $50 ticket for a trip to Wisconsin. You think you will enjoy the Wisconsin trip more than the Michigan trip. When you are putting your just-purchased Wisconsin trip ticket in your wallet*, *you notice that the Michigan trip and the Wisconsin trip are for the same weekend! It is too late to refund either ticket. You must use one ticket and not the other. Which trip will you go on?”*^1^.

According to an assumption of traditional economics theory, decisions should be made based on the costs and benefits expected to arise in the future from the choice of each option^4^. Based on this assumption, everyone would be expected to choose the trip considered to be more enjoyable (i.e., the trip to Wisconsin). However, about half of the participants in the experiment chose the Michigan trip instead, which had incurred the larger sunk cost^1^. Because an invested sunk cost cannot be recovered, a rational forward-looking decision maker should ignore sunk costs^1^,^2^,^3^. However, nonhuman species, individuals, companies, and even governments often decide without ignoring sunk costs. For example, people who pay less at an all-you-can-eat pizza restaurant, because of a surprise reduction in the price, then eat less pizza^5^. Singaporeans who happen to pay more for an expensive car permit then drive their cars more^6^. These types of maladaptive behavior can also lead to severe financial or political consequences such as continuation of an unprofitable building project or war^1^,^2^,^3^,^7^. Please see Supplementary Introduction for details regarding prior research concerning the sunk cost effect.

This leads to a question intended to prevent serious consequences: who is more vulnerable to the sunk cost effect? Although some factors were reported to be associated with the sunk cost effect^8^,^9^, this issue remains largely unknown. Such differences are important because they are clues to the psychological mechanism generating the effect, and for placing the right type of people in organizational jobs when ignoring sunk cost is beneficial.

Meanwhile, our understanding of how decision-making is implemented in the brain has grown rapidly in recent years^10^,^11^,^12^,^13^,^14^,^15^. Although the neural bases of many types of decision-making have been extensively examined, to the best of our knowledge, only two neuroimaging studies have focused on decisions involving sunk costs^16^,^17^; those studies are described in the Discussion section below. Neither of these studies measured participants’ personality traits, and therefore the neural mechanism that underlies individual differences in the sunk cost effect remains unclear.

Here, we combined functional magnetic resonance imaging (fMRI) and an assessment of the core aspects of human personality. Previous neuroeconomics studies repeatedly reported that emotions play essential roles in decision-making processes, and that individual differences in neural activity of the emotion-related brain regions [e.g., insula, amygdala, anterior cingulate cortex (ACC)] explain susceptibility to a biased and irrational decision-making^18^,^19^,^20^. In line with this notion, a previous behavioral study showed that the negative emotions associated with sunk costs could bias decision-making under sunk costs^21^. Accordingly, one might intuitively predict that individuals sensitive to negative emotions or stress (e.g., people with high neuroticism) would be more susceptible to a sunk cost effect. However, sunk costs may elicit strong negative emotions in people who feel fear or a sense of guilt regarding violations of the “don’t waste” rule. In other words, it could be predicted that people who adhere to social norms and values are more susceptible to the sunk cost effect. Furthermore, we hypothesized that this potential observation might be explained by the elevated activation in brain regions crucial to negative emotional processing during decision-making under sunk costs.

## Results

**Figure 1:**
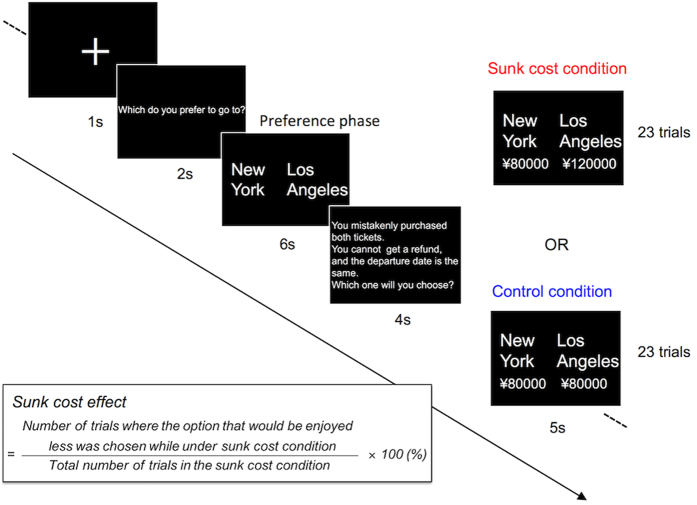
Experimental design. In each trial, subjects first express a preference for which of two cities to travel to (preference phase). Then they are told they have, by mistake, previously paid for nonrefundable tickets to both cities (which are sunk costs). In the control condition the tickets are priced reasonably and the costs of the two tickets are identical. In the sunk cost condition the ticket for the non-preferred destination is 50% higher than the ticket price from the preferred destination. A decision maker sufficiently influenced by the sunk cost will switch his/her choice in this condition. Figure 1 depicts the case in which New York is chosen in the preference phase. Estimation of the sunk cost effect is also described.

We modified a clear example of the sunk cost effect described above (Fig. 1). Subjects first express a preference for which of two cities to travel to. Then they are told they have, by mistake, previously paid for nonrefundable tickets to both cities (which are sunk costs). In the control condition the tickets are priced reasonably and the costs of the two tickets are identical. In the sunk cost condition the ticket for the non-preferred destination is 50% higher than the ticket price from the preferred destination. A decision maker sufficiently influenced by the sunk cost will switch his/her choice in this condition.

### Behavior data

Participants (*N* = 32) performed the task well, only missing an average of 0.53 (*S*.*D*. = 0.67) trials (46 trials in total). The mean rates of choosing the option that would be enjoyed less were 47.9 (*S*.*D*. = 35.5) % under the sunk cost condition but only 0.8 (*S*.*D*. = 2.0) % under the control condition, which indicates that the participants’ decisions were influenced by sunk costs.

**Figure 2:**
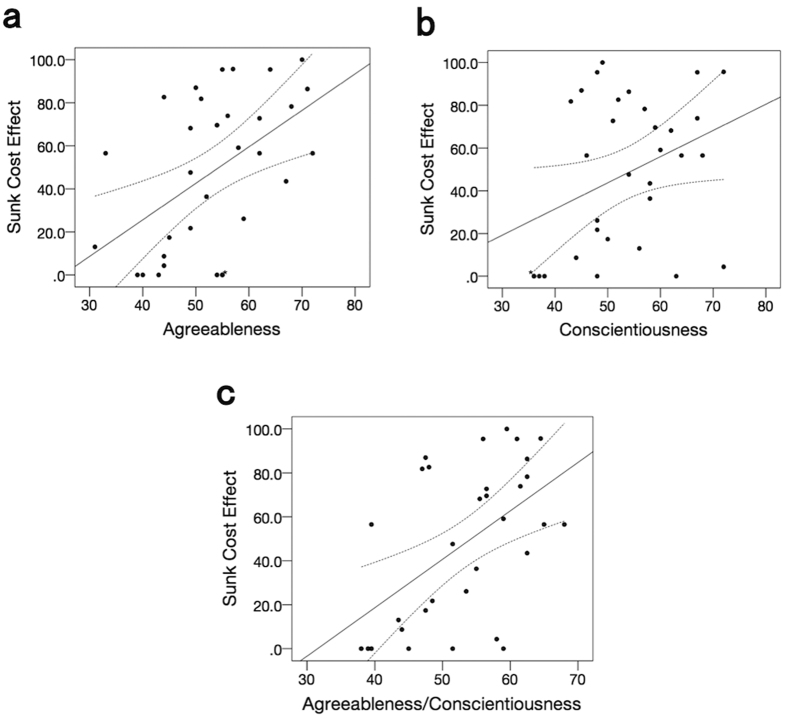
Correlation between personality traits and the sunk cost effect. Plots and regression lines of correlation between personality traits and the sunk cost effect are shown (*N* = 32). (**a**) Correlation between agreeableness and the sunk cost effect (*r* = 0.51, *p* = 0.003), (**b**) correlation between conscientiousness and the sunk cost effect (*r* = 0.36, *p* = 0.046), and (**c**) correlation between averaged score of these two personality traits and the sunk cost effect (*r* = 0.53, *p* = 0.002). Dashed lines are 95% confidence interval boundaries. *overlapping data point.

In the five domains of the Revised NEO Personality Inventory (NEO-PI-R)^22^,^23^, the measures of both agreeableness and conscientiousness were positively correlated with individual differences in the strength of the sunk cost effect (*r* = 0.51, *p* = 0.003 and *r* = 0.36, *p* = 0.046, respectively, Fig. 2a,b); the other three domains did not significantly correlate with the sunk cost effect (all, *p* > 0.31, Supplementary Table S1). The results of each domain in NEO-PI-R were provided in Supplementary Table S2. Among the five domains of NEO, high levels of agreeableness and conscientiousness have both been reported to associate with adherence to social rules and decreased risk taking^24^,^25^, whereas higher scores in neuroticism, extraversion, and openness are associated with increased risk taking^24^,^26^. Therefore, we also averaged these two scores (agreeableness, conscientiousness) and correlated that average with the sunk cost effect. The averaged score was more strongly correlated with the sunk cost effect (*r* = 0.53, *p* = 0.002, Fig. 2c).

### fMRI data

**Figure 3:**
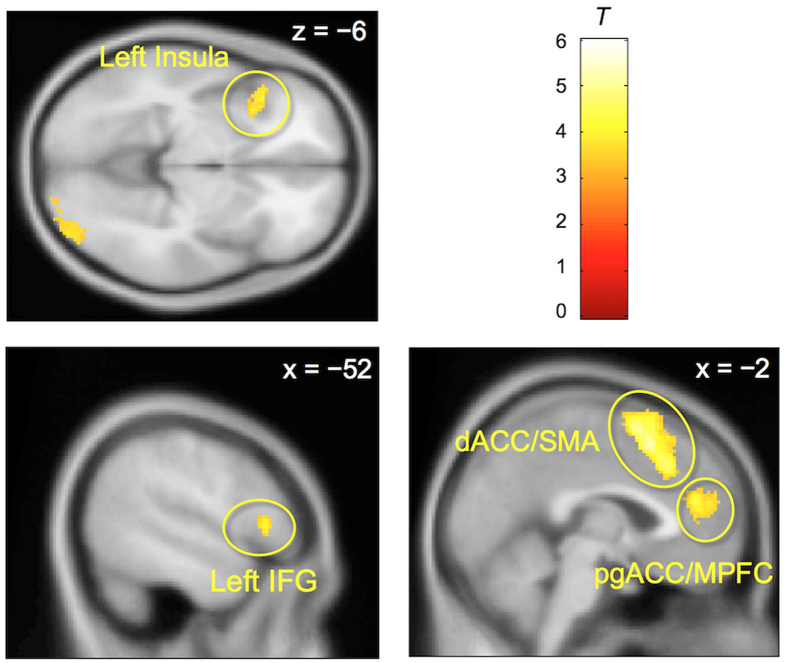
Activations associated with decision-making under sunk costs. A statistical threshold was set at cluster-level FDR corrected *q* < 0.01 (*N* = 32). dACC = dorsal anterior cingulate cortex, IFG = inferior frontal gyrus, MPFC = medial prefrontal cortex, pgACC = pregenual anterior cingulate cortex, SMA = supplementary motor area.

**Figure 4:**
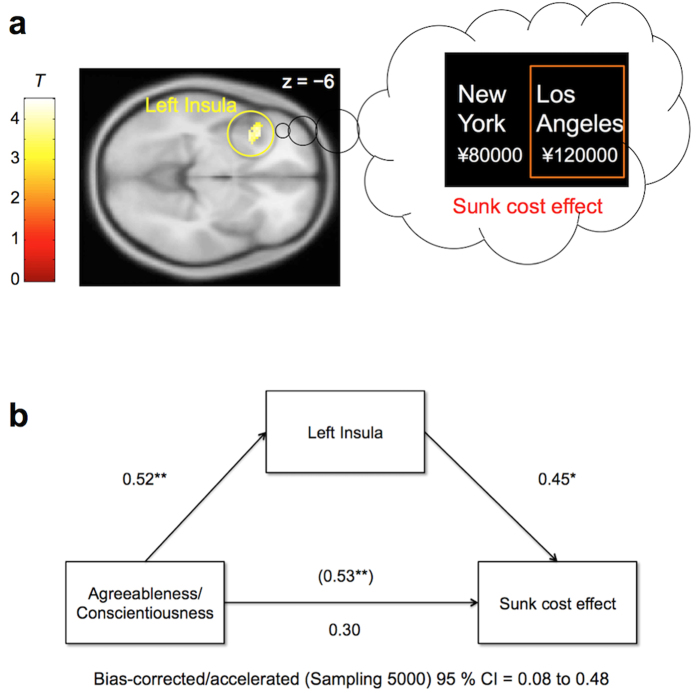
Contribution of the insula in the sunk cost effect. (**a**) The cluster including the left insula (peak MNI coodinates: –32, 16, –10) was positively correlated with individual differences in the sunk cost effect (cluster-level FDR corrected *q* < 0.05, *N* = 32). (**b**) Bias-corrected/accelerated 95% confidence intervals (CI) for the indirect effect from a bootstrap-mediation analysis found that activation in the left insula mediated the relationship between the averaged score of agreeableness and conscientiousness and the sunk cost effect (*N* = 32). Standardized coefficients and significance indicated by asterisks are reported for each path. *p < 0.05, **p < 0.01.

Figure 3 and Supplementary Table S3 present the neural activations associated with decision-making under sunk costs (i.e., sunk cost condition >control condition). We identified several regions of activation, including the left insula, inferior frontal gyrus (IFG), pregenual ACC (pgACC), dorsal ACC (dACC) extending into the supplementary motor area (SMA), and right occipital lobe. In contrast, we did not find any significant regions of deactivation on decision-making under sunk costs (i.e., control condition >sunk cost condition) at the same thresholds. Of these identified areas of differential activation, only the levels of activation in the left insula (positively) correlated with the sunk cost effect (Fig. 4a and Supplementary Table S4). This correlation was not evident in other areas such as the ACC. There were no brain areas with activation showing significant negative correlation with the sunk cost effect.

Next, we conducted mediation analyses to examine whether this activation mediated the relationship between the identified personality traits and the sunk cost effect. These analyses revealed that activation in the left insula mediated the relationship of these personality traits (agreeableness, conscientiousness, and averaged scores of these personality domains) with the sunk cost effect (Fig. 4b and Supplementary Fig. S1).

**Figure 5:**
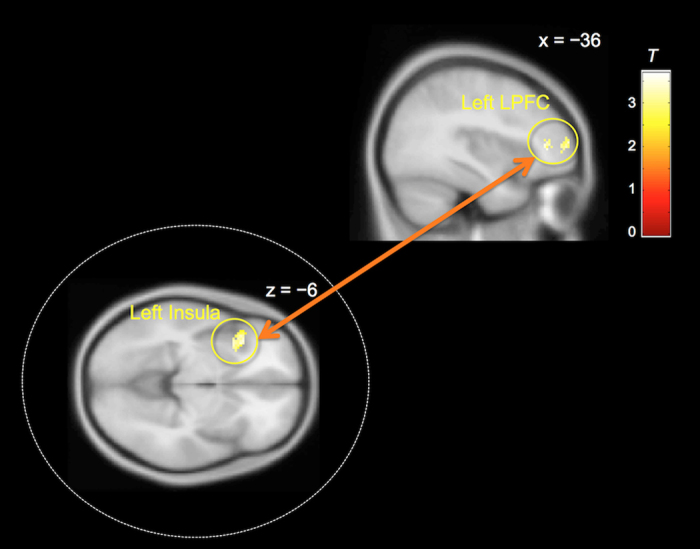
Result of psychophysiological interaction (PPI) analysis. The functional connectivity between the left insula and left lateral prefrontal cortex (LPFC) was increased during decision-making under sunk costs (cluster-level FDR corrected *q* < 0.05, *N* = 32).

Finally, we performed psychophysiological interaction (PPI) analysis using the left insula as seed region. The results showed that the functional connectivity between the left insula and left lateral prefrontal cortex (LPFC), including the IFG, was increased during decision-making under sunk costs (Fig. 5 and Supplementary Table S5). We did not find any significant regions showing decreased functional connectivity with the left insula at the same threshold.

### Additional analyses

Because some participants showed preference reversals in some trials of the control condition, we also analyzed the data using the sunk cost measure taking this into account (rates of preference reversals under sunk cost condition – those under control condition). The results did not influence our conclusion (please see Supplementary Results, Supplementary Fig. S2 and Table S6 for details).

## Discussion

We found that individuals with high levels of agreeableness and conscientiousness were more susceptible to the sunk cost effect. Furthermore, we show that elevated neural activation in the insula explains the relationship between these personality traits and the sunk cost effect. Our findings comprise important practical implications and add to our understanding of the psychological and neural mechanisms of this effect.

Agreeableness is a personality trait characterized by altruism, compliance, and tender-mindedness. Highly agreeable people are well socialized and avoid conflict in social situations^22^,^27^. Individuals with high levels of conscientiousness are organized, careful, reliable, hard-working, and avoid counterproductive work behaviors^22^,^28^. Among the five domains of NEO, higher scores in agreeableness and conscientiousness have been reported to associate with a reduction in risk taking behaviors^24^,^25^, whereas higher scores in neuroticism, extraversion, and openness are associated with increased risk taking^24^,^26^. In other words, individuals with high levels of agreeableness and conscientiousness are people who tend to strongly adhere to social norms and values. Notably, however, the present results suggest that these people, who are adaptive in normal daily life, may paradoxically make maladaptive decisions under sunk cost situations. Our finding may offer clues for the possible psychological mechanism generating the sunk cost effect.

The fMRI showed a stronger activation in the left insula during decision-making under sunk costs. Furthermore, differential activity in the same region across subjects was highly correlated with individual differences in the strength of the sunk cost effect. These results suggest that the insula is a key neural substrate underlying individual differences in the sunk cost effect.

Further analyses showed that this insula activity mediated the relationship between the agreeableness/conscientiousness and the sunk cost effect. The insula is known to be involved in olfaction, gustation, pain, and somatosensory processing^29^. Especially, the dorsal anterior region identified in our study plays an essential role in negative emotional processing, and activity there has therefore been proposed as a neural marker of anticipatory negative affect^30^,^31^,^32^. We speculate that sunk costs may elicit stronger negative emotions in individuals socialized to follow rules and regulations (those with high levels of agreeableness/conscientiousness), and as a result such people experience greater sunk cost effects. In addition, previous studies have reported that people exhibiting higher insula activation during risky decision-making exhibited higher loss avoidance; the authors proposed that this region plays a key role in learning to avoid losses^33^,^34^. Taken together, our findings support the theory that the sunk cost effect may, at least partially, be due to adherence to a no-waste principle^1^,^2^.

Contrary to the intuitive prediction that a personality sensitive to negative emotions (neuroticism) would be related to the sunk cost effect, we found no correlation between the levels of neuroticism and the sunk cost effect. One possible interpretation is that, as mentioned above, sunk costs might elicit strong negative emotions in people who feel fear or a sense of guilt regarding violations of a no-waste principle. This notion should be further investigated in future studies.

The results of the functional connectivity analysis also supported that the insula is a key structure involved in the sunk cost effect. The PPI analysis showed that the functional connectivity between the left insula and left LPFC, including the IFG, was enhanced during decision-making under sunk costs. The LPFC is known to be involved in self-control^35^, emotion regulation^36^, and working memory^37^,^38^. Furthermore, social norms are thought to be represented in the LPFC, and this area plays a key role in rule-based control^39^,^40^. In line with this notion, a recent study showed that anodal transcranial direct current stimulation (tDCS) of dorsal LPFC increases the sunk cost effect^41^. Therefore, tight coupling between these two regions might bias decision-making under the influence of sunk costs.

In addition to the left insula and the IFG, we found that the pgACC/MPFC and dACC/SMA were activated during decision-making under sunk costs. These ACC subregions are known to be involved in emotion regulation^42^ and cognitive/emotional conflict processing^43^,^44^. The cluster we identified (peak coordinates; −6, 38, 20) also partly overlaps with the brain areas that have been associated with goal valuation in previous studies^35^,^45^. Our results may suggest that the decision regarding whether to prioritize preference or sunk costs involves an emotional conflict. Of course, this is a post-hoc inference mentioned to guide future work and is clearly speculative.

Two previous studies explored neural activity involving sunk costs^16^,^17^. Both studies used a project-continuation paradigm. In this paradigm, an initial investment is made but project completion requires a further incremental investment. The sunk cost effect was shown because people were more willing to make the second incremental investment if a previous investment was made. However, sunk costs are not restricted to situations with negative feedback and uncertainty of decision consequences^1^. Thus, it is important to apply a variety of sunk cost scenarios for a better understanding of the neural mechanisms of decision-making under sunk costs. In this study, we employed a different example of the sunk cost effect from that used in a previous study^1^. The neural results of the current study partly differ from those of previous studies, which might be due to difference of the experimental paradigms applied.

One study^16^ found distinct neural coding of the sunk cost and the incremental cost, with coding of sunk cost in parietal and frontal areas (overlapping with the SMA area in our Fig. 3). However, the authors did not associate neural activity and behavior. The second study^17^ found more activity in the dorsal LPFC and amygdala when sunk costs were larger; they also found activity in ACC as we did. Across subjects, the sunk cost effect is only weakly correlated positively with dorsal LPFC activity. However, a direct individual-specific measure of desire to avoid waste is more strongly correlated with dorsal LPFC (most recently, the same group again showed a critical role of the dorsal LPFC in the sunk cost effect using transcranial direct current stimulation^41^). Our study is consistent with the latter finding, that following a no-waste principle causes the sunk cost effect, and lateral PFC areas are associated with implementation of this principle.

There are several limitations to this study. First, in the current task, the ticket prices were systematically higher in the sunk cost condition compared with the control condition because the ticket price for the non-preferred destination was presented at 50% higher than the ticket price from the preferred one in the sunk cost condition. It is possible that brain activation during sunk cost decision-making identified in the current study could have partially reflected higher incurred ticket prices of the non-preferred option or the higher sum of the preferred and non-preferred ticket prices. However, even reducing the confounding effect of the ticket prices, we still found that our identified regions (e.g., insula, IFG, ACC) were activated, indicating the crucial roles of these areas in sunk cost decision-making (please see Supplementary Results and Supplementary Table 7). Second, this study used a hypothetical choice experiment to understand actual choices under the influence of sunk costs. Further research implementing real choice will be needed to support the present results. Finally, we recruited our sample from graduate and undergraduate students to ensure a uniform socioeconomic status among our participants. Therefore, our findings might not be representative of various samples with different backgrounds and socioeconomic status levels. Our results should be generalized cautiously. Notwithstanding these limitations, our findings revealed how individual differences can affect decision-making under sunk costs, thereby contributing to a better understanding of the psychological and neural mechanisms of the sunk cost effect.

## Methods

### Participants

Thirty-five healthy right-handed volunteers participated in this study. They were recruited from among undergraduate and graduate students (three were research fellows or trainees). The sample size was determined based on the previous neuroimaging studies of individual differences in decision-making e.g., refs 34,46. The volunteers did not meet the criteria for any psychiatric disorders according to the Structured Clinical Interview for DSM-IV Axis I Disorders (SCID I). They also did not have a history of head trauma, any neurological illness, serious medical or surgical illness, or substance abuse. Predicted IQ was estimated using the Japanese Version of the National Adult Reading Test short form^47^. Three participants were excluded from the analyses: one exhibited insufficient responses during the task [she did not make a response in 15 out of the 46 trials, which was extremely high as compared to the average missed trials of the participants (>2 *S*.*D*. + mean)]. Two participants showed excessive head motion (>3 mm) during the scanning. Therefore, we analyzed data from the remaining 32 participants [12 females; mean age: 24.5 (*S*.*D*. = 6.5) years (range 19–38 years)]. Estimated mean IQ was 107.6 (*S*.*D*. = 7.3).

This study was approved by the Committee on Medical Ethics of Kyoto University and was carried out in accordance with The Code of Ethics of the World Medical Association. After complete description of the study, written informed consent was obtained from each participant.

### fMRI Task

We modified the sunk cost task used in a previous study^1^. Participants were initially instructed to select their preferred option from two travel destinations by pressing a button on a magnet-compatible button-box (preference phase). Next, participants received the following instructions: “You mistakenly purchased both of the tickets. You cannot get a refund, and the departure date is the same for both. Which one will you choose”? The task had 46 trials. In half of the trials (control condition), the costs of the two tickets were identical. Twenty-three different pairs of destinations (e.g., New York vs Los Angeles) were presented. Each destination was presented only once (all 23 destination pairs are shown in Supplementary Table S8). The costs of the tickets were defined realistically, from among five costs (¥30,000, ¥40,000, ¥50,000, ¥80,000, ¥100,000). In the other half of the trials (sunk cost condition), the participants selected from the same destination pairs and ticket costs. However, tickets for the destinations non-preferred (not chosen by the participants in the preference phase) were presented as 1.5 times more expensive than tickets for the preferred (chosen in the preference phase) destinations. A decision maker sufficiently influenced by the sunk cost would switch his/her choice in this condition. The trial order was pseudo-randomized and the total length of the task was 13 min and 57 s. The time course is shown in Fig. 1. The experiment was conducted using E-Prime software (Psychology Software Tools, Inc., Pittsburgh, PA, USA).

### Personality assessment

We administered the Japanese version of NEO-PI-R^22^,^23^, one of the most widely used questionnaires for assessing broad personality traits. This self-report questionnaire comprises 240 items and contains five dimensional scales (neuroticism, extraversion, openness, agreeableness, and conscientiousness) that correspond to a five-factor personality trait model. Neuroticism consists of 48 items (e.g., “I am easily frightened”), extraversion has 48 items (e.g., “I like to have a lot of people around me”), openness has 48 items (e.g., “I have a very active imagination”), agreeableness has 48 items (e.g., “I would rather cooperate with others than compete with them”), and conscientiousness also has 48 items (e.g., “I’m known for my prudence and common sense”). Participants are asked to respond to each item by designating answers from 0 (strongly disagree) to 4 (strongly agree). NEO-PI-R results are presented as T scores with a mean of 50 and standard deviation (*S*.*D*.) of 10. Higher scores indicate higher levels of the particular trait.

### fMRI data acquisition and pre-processing

fMRI images were scanned on a 3 T Trio (Siemens, Erlangen, Germany) equipped with an 8-channel phased-array head coil and pre-processed using SPM8 (Wellcome Trust Center for Neuroimaging, London, UK) (see Supplementary Methods for a more detailed description of the fMRI data acquisition and pre-processing).

### Statistical analyses

#### Demographic and behavioral data (sunk cost effect)

Demographic and behavioral data were analyzed using SPSS 21 (IBM, Armonk, NY, USA). We estimated the participants’ sunk cost effects by calculating the rate of choosing the option that would be enjoyed less (i.e., was initially dispreferred, but has a higher sunk ticket price) while under sunk cost condition (Fig. 1). We performed Pearson correlation analyses between the sunk cost effect and each T score of the five domains of NEO-PI-R to investigate the relationship between the sunk cost effect and the core aspects of human personality. Furthermore, because high levels of agreeableness and conscientiousness were both reported to associate with adherence to social rules and decreased risk taking^24^,^25^, we averaged these two scores and performed correlation analysis with the sunk cost effect (please see Results section). Results were considered statistically significant at *p* < 0.05 (two-tailed).

### fMRI data

After pre-processing, we fitted a general linear model (GLM)^48^ to the fMRI data. In the first-level analyses, the design matrix contained three task-related regressors (preference phase, and sunk cost and control conditions). We used the time-point of the button press as onset (duration, 0 s) based on the recent fMRI studies of decision-making e.g., ref. 17. To minimize motion-related artifacts, six movement parameters (three displacements and three rotations) were also included as additional regressors of no interest. Data were high-pass filtered at 128 s. The activation associated with decision-making under sunk costs was identified as the difference between the sunk cost and control conditions. The comparison produced a contrast image for each participant, which was used for the second-level fMRI analyses.

In the second-level analyses, we used a random-effects model to draw population-level inferences. First, the main effects of decision-making under sunk costs were computed using one-sample t-tests (whole-brain analysis). Clusters that survived the false-discovery rate (FDR) correction for multiple comparisons with a cluster-level *q* < 0.01 (at voxel-level uncorrected *p* < 0.001) were reported.

Next, we performed a voxelwise regression analysis using the above-mentioned clusters as an inclusion mask to explore the brain regions in which activity during decision-making under sunk costs correlated with individual differences in the sunk cost effect. The statistical threshold was defined at a cluster-level *q* < 0.05 after correcting for multiple comparisons using FDR (at voxel-level uncorrected *p* < 0.005). The VOI function in SPM8 was used to extract the parameter estimates from the significant clusters. Next, we conducted mediation analyses using the INDIRECT macro for SPSS^49^ to investigate the mediation effect of neural activation in areas where correlations were observed for the relationships between personality traits and the sunk cost effect. We tested the significance of the mediation effect with a bootstrapping strategy within this macro. Using this strategy, if a confidence interval does not contain zero, the mediated effect is considered significant^49^.

Finally, we conducted PPI analysis^50^ to identify areas in the brain that showed significant co-variation with the brain region that correlated with individual differences in the sunk cost effect (whole-brain analysis). The above-mentioned analyses identified that the left insula (peak MNI coodinates: −32, 16, −10) was correlated with individual differences in the sunk cost effect, and thus we selected this region as a seed region. Firstly, the first eigenvariate time series for each participant was extracted from the left insula (centered at −32, 16, −10; with a 5-mm radius). The PPI was then calculated as the product of the time series of the seed region and a vector coding for the psychological context (i.e., the sunk cost – control condition). For each participant, we created a new GLM including the PPI as a regressor together with the physiological and psychological vectors. The GLM also included six movement parameters as regressors of no interest. The participants’ specific contrast images were then entered into random effects group analyses. The statistical threshold was defined at a cluster-level *q* < 0.05 after correcting for multiple comparisons using FDR (at voxel-level uncorrected *p* < 0.005).

We interpreted the anatomical locations of clusters by consulting the Talairach Daemon database (http://www.talairach.org), the Anatomic Automatic Labeling toolbox^51^, and neuroanatomy atlas books^52^,^53^.

## Additional Information

**How to cite this article**: Fujino, J. *et al.* Neural mechanisms and personality correlates of the sunk cost effect. *Sci. Rep.* **6**, 33171; doi: 10.1038/srep33171 (2016).

## Supplementary Material

Supplementary Information
