# Supplementary material for: Neural mechanisms and personality correlates of the sunk cost effect
Source: Sci Rep. 2016 Sep 9;6:33171. doi: 10.1038/srep33171 (PMC5017311; doi:10.1038/srep33171)
Supplement: Supplementary Information [file srep33171-s1.pdf]

**Supplementary Information**

# Neural mechanisms and personality correlates of the sunk cost effect

Junya Fujino[1,2,†], Shinsuke Fujimoto[1,†], Fumitoshi Kodaka[3], Colin F. Camerer[4], Ryosaku Kawada[1], Kosuke Tsurumi[1], Shisei Tei[1,5,6], Masanori Isobe[1], Jun Miyata[1], Genichi Sugihara[1], Makiko Yamada[7], Hidenao Fukuyama[8], Toshiya Murai[1], Hidehiko Takahashi[1,*]

[1]Department of Psychiatry, Kyoto University Graduate School of Medicine

[2]Medical Institute of Developmental Disabilities Research, Showa University

[3]Department of Psychiatry, The Jikei University School of Medicine

[4]Division of Humanities and Social Sciences, California Institute of Technology

[5]Institute of Applied Brain Sciences, Waseda University

[6]School of Human and Social Sciences, Tokyo International University

[7]Molecular Neuroimaging Program, Molecular Imaging Center, National Institute of Radiological Sciences

[8]Human Brain Research Center, Kyoto University Graduate School of Medicine

[†]**These authors contributed equally to this work.**

[*]**Corresponding author:** Hidehiko Takahashi, MD, PhD

Department of Psychiatry, Kyoto University Graduate School of Medicine, 54 Shogoin-Kawaracho, Sakyo-ku, Kyoto 606-8507, Japan

Tel: +81-75-751-3386, Fax: +81-75-751-3246, E-mail: hidehiko@kuhp.kyoto-u.ac.jp

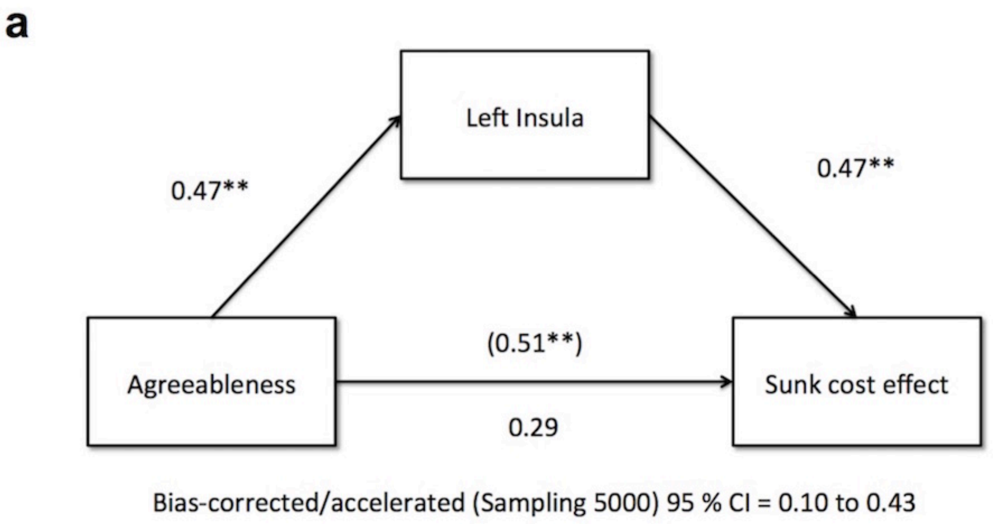


**Supplementary Figure S1.**

**Results of mediation analyses.** Bias-corrected/accelerated 95% confidence intervals (CI) for the indirect effect from a bootstrap-mediation analysis found that activation in the left insula mediated both the relationship between the agreeableness domain and the sunk cost effect (a) and the relationship between the conscientiousness domain and the sunk cost effect (b). Standardized coefficients and significance indicated by asterisks are reported for each path ($N = 32$). $*p < 0.05$, $**p < 0.01$

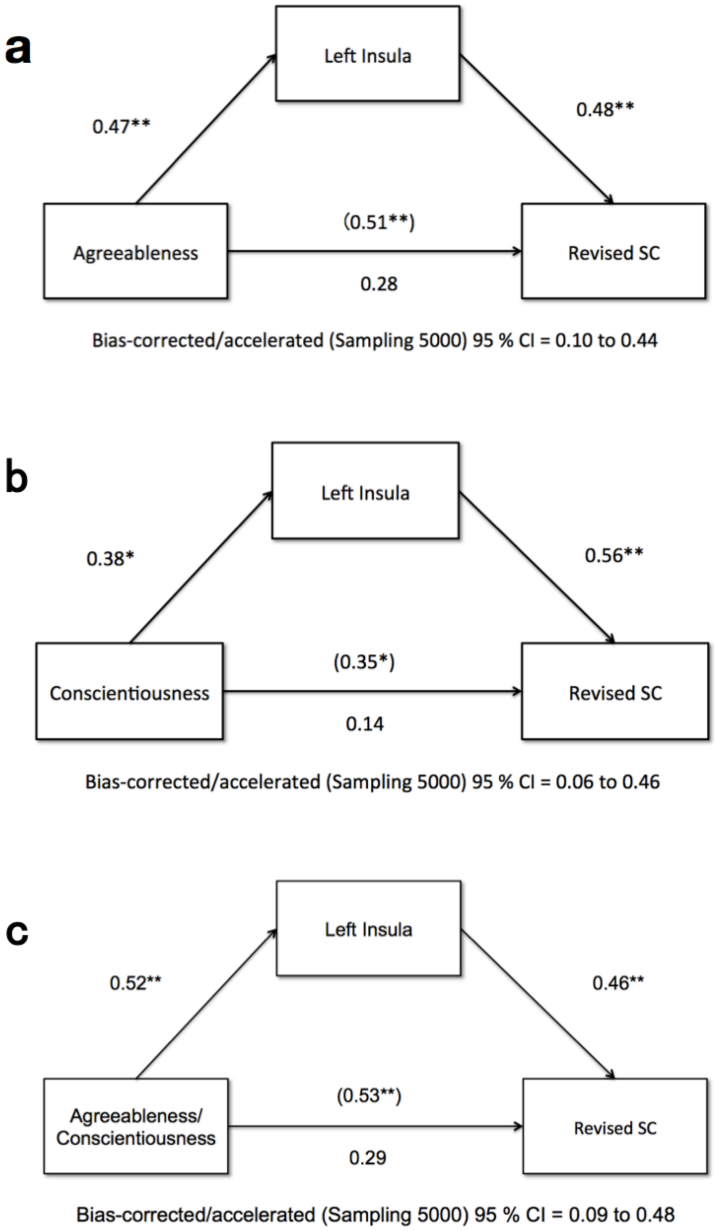


**Supplementary Figure S2.**

**Results of mediation analyses [revised sunk cost measure (revised SC)].**

Bias-corrected/accelerated 95% confidence intervals (CI) for the indirect effect from a bootstrap-mediation analysis found that activation in the left insula mediated the relationship between the agreeableness domain and the revised SC (a), the relationship between the conscientiousness domain and the revised SC (b), and the relationship between the averaged score of agreeableness and conscientiousness and the revised SC (c). Standardized coefficients and significance indicated by asterisks are reported for each path ($N = 32$). $^{*}p < 0.05$, $^{**}p < 0.01$

| | Sunk cost effect |
|---|---|
| Neuroticism | $r = -0.18$ ($p = 0.32$) |
| Extraversion | $r = 0.07$ ($p = 0.71$) |
| Openness | $r = 0.08$ ($p = 0.68$) |
| Agreeableness | $r = 0.51$ ($p = 0.003$)** |
| Conscientiousness | $r = 0.36$ ($p = 0.046$)* |

**Supplementary Table S1.**

**Correlation coefficient between the 5 domains in NEO and the sunk cost effect**

*$p < 0.05$, **$p < 0.01$

| | Mean ± S.D. |
|---|---|
| Neuroticism | 48.4 ± 11.1 |
| Extraversion | 48.1 ± 11.2 |
| Openness | 48.7 ± 8.5 |
| Agreeableness | 53.2 ± 10.6 |
| Conscientiousness | 53.4 ± 10.3 |

**Supplementary Table S2.**

**Results of each domain in NEO-PI-R**

| Brain Region | Coordinates (mm) | | | t | Cluster |
|---|---|---|---|---|---|
| | x | y | z | | (voxels) |
| L SMA | −6 | 6 | 62 | 6.00 | 2016 |
| | −2 | 16 | 52 | 5.30 | |
| L dACC | −4 | 24 | 40 | 5.20 | |
| L pgACC | −6 | 38 | 20 | 5.02 | 481 |
| | −8 | 44 | 12 | 4.27 | |
| L MPFC | −6 | 52 | 16 | 4.03 | |
| L insula | −30 | 16 | −12 | 4.59 | 553 |
| L IFG | −48 | 24 | 4 | 4.42 | |
| | −32 | 24 | −16 | 4.41 | |
| R occipital lobe | 32 | −96 | 4 | 4.07 | 326 |
| | 40 | −92 | 2 | 4.01 | |
| | 42 | −84 | −8 | 3.91 | |

**Supplementary Table S3.**

**Activations associated with decision-making under sunk costs.**

$q < 0.01$ (cluster-level FDR corrected)

Three local maxima more than 8.0mm apart are reported.

The coordinates are in the MNI space.

Abbreviations: dACC = dorsal anterior cingulate cortex, FDR = false discovery rate, IFG = inferior frontal gyrus, L = left, MNI = Montreal Neurological Institute, MPFC = medial prefrontal cortex, pgACC = pregenual anterior cingulate cortex, SMA = supplementary motor area, R = right

| Brain Region | Coordinates (mm) | | | t | Cluster |
|---|---|---|---|---|---|
| | x | y | z | | (voxels) |
| L insula | −32 | 16 | −10 | 4.50 | 230 |
| | −34 | 24 | −2 | 4.32 | |
| | −42 | 16 | −14 | 3.60 | |

**Supplementary Table S4.**

**Activations associated with the sunk cost effect.**

$q < 0.05$ (cluster-level FDR corrected)

Three local maxima more than 8.0mm apart are reported.

The coordinates are in the MNI space.

Abbreviations: FDR = false discovery rate, L = left, MNI = Montreal Neurological Institute

| Brain Region | Coordinates (mm) | | | t | Cluster |
|---|---|---|---|---|---|
| | x | y | z | | (voxels) |
| L SFG | −18 | 58 | 24 | 3.68 | 364 |
| L IFG | −48 | 42 | 12 | 3.64 | |
| L SFG | −12 | 60 | 16 | 3.59 | |

**Supplementary Table S5.**

**Results of psychophysiological interaction (PPI) analysis**

$q < 0.05$ (cluster-level FDR corrected)

Three local maxima more than 8.0mm apart are reported.

The coordinates are in the MNI space.

Abbreviations: FDR = false discovery rate, IFG = inferior frontal gyrus, L = left, MNI = Montreal Neurological Institute, SFG = superior frontal gyrus

| | Sunk cost effect |
|---|---|
| Neuroticism | $r = -0.19$ ($p = 0.31$) |
| Extraversion | $r = 0.07$ ($p = 0.69$) |
| Openness | $r = 0.07$ ($p = 0.69$) |
| Agreeableness | $r = 0.51$ ($p = 0.003$)** |
| Conscientiousness | $r = 0.35$ ($p = 0.046$)* |

**Supplementary Table S6.**

**Correlation coefficient between the 5 domains in NEO and the revised SC**

*$p < 0.05$, **$p < 0.01$

| Brain Region | Coordinates (mm) | | | t | Cluster (voxels) |
|---|---|---|---|---|---|
| | x | y | z | | |
| L MPFC | −8 | 58 | 16 | 5.48 | 207 |
| L ACC | −6 | 42 | 16 | 3.92 | |
| | −6 | 38 | 24 | 3.44 | |
| L SFG | −14 | 42 | 50 | 5.33 | 926 |
| L SMA | −8 | 6 | 68 | 4.89 | |
| L MPFC | −10 | 28 | 62 | 4.71 | |
| L insula | −30 | 16 | −12 | 4.34 | 241 |
| L IFG | −32 | 24 | −16 | 4.21 | |
| | −36 | 22 | −4 | 4.17 | |

**Supplementary Table S7.**

**Results of additional analyses concerning activation associated with decision-making under sunk costs**

$q < 0.05$ (cluster-level FDR corrected)

Three local maxima more than 8.0mm apart are reported.

The coordinates are in the MNI space.

One cluster [bilateral occipital lobe (peak coordinates: −10, −76, −6, $t = 4.30$, $k = 479$)] was observed in the contrast “control condition > sunk cost condition” with the same threshold (cluster-level FDR corrected $q < 0.05$).

Abbreviations: ACC = anterior cingulate cortex, FDR = false discovery rate, IFG = inferior frontal gyrus, L = left, MNI = Montreal Neurological Institute, MPFC = medial prefrontal cortex, SFG = superior frontal gyrus, SMA = supplementary motor area

| **Pairs of destinations** | |
|---|---|
| London (¥100,000) | Manchester (¥100,000) |
| San Francisco (¥100,000) | Las Vegas (¥100,000) |
| Tahiti (¥50,000) | Bali (¥50,000) |
| Macau (¥30,000) | Hong Kong (¥30,000) |
| Shanghai (¥40,000) | Beijing (¥40,000) |
| Berlin (¥100,000) | Munich (¥100,000) |
| Seoul (¥30,000) | Taipei (¥30,000) |
| Monaco (¥50,000) | Sicily (¥50,000) |
| Florence (¥100,000) | Venice (¥100,000) |
| Saipan (¥50,000) | Guam (¥50,000) |
| Rome (¥100,000) | Milan (¥100,000) |
| Moscow (¥100,000) | Saint Petersburg (¥100,000) |
| Toronto (¥80,000) | Montreal (¥80,000) |
| Sao Paulo (¥100,000) | Rio (¥100,000) |
| Sydney (¥80,000) | Melbourne (¥80,000) |
| New York (¥80,000) | Los Angeles (¥80,000) |
| Paris (¥100,000) | Nice (¥100,000) |
| Kamakura* (¥30,000) | Hakone* (¥30,000) |
| Izu* (¥40,000) | Atami* (¥40,000) |
| Otaru* (¥30,000) | Hakodate* (¥30,000) |
| Beppu* (¥40,000) | Noboribetsu* (¥40,000) |
| Yakushima* (¥40,000) | Awami* (¥40,000) |
| Niigata* (¥30,000) | Hokkaido* (¥30,000) |

**Supplementary Table S8.**

**All 23 pairs of destinations in the fMRI task**

The defined costs of the tickets in the control condition are provided in parentheses.

*Popular travel destinations in Japan

## Supplementary Introduction

### Prior research concerning the sunk cost effect

This section briefly describes previous empirical evidence regarding the sunk cost effect.

The first discussion of sunk cost effects came from organizational behavior scholars. They demonstrated in hypothetical lab experiments that subjects were more likely to continue investing in a project if its previous (sunk) costs were high[1]. This type of decision came to be called a "progress" decision. An influential 1980 paper described sunk cost effects in a different type of "utilization" decision: Suppose you bought tickets for a basketball game and a snowstorm arose. Many people would go because they don't want to "waste" the tickets. But if they hadn't bought the tickets yet, or lost the ticket and had to replace it, they would not go or buy a new one[2]. (The utilization paradigm is used in our study.) Subsequent studies dissected the possible causes of the sunk cost effect in different experiments[3]. Most experiments find sunk cost effects, though effects are often small in magnitude and not completely robust to treatment changes[4].

In establishing the sunk cost effect, it is extremely important to control for perceptions of any marginal costs (e.g. from investing further in progress decisions) and marginal benefits[5,6]. For example, suppose a company builds a building and expects it to cost \$10 million, and values it at \$15 million. After finishing half of the building, spending \$5 million, they realize it will cost \$12 million more to complete it. Suppose the half-finished building is worthless unless it is finished. Then the company must decide whether to spend an additional \$12 million to have a building worth \$15 million.

The company *should* continue to spend money because the marginal benefit minus the marginal cost is positive (=15-12=+3). When they are done, they will have paid $17 million for a building worth $15 million. But the mistake that was made was underestimating the chance of a cost overrun. Paying the marginal $12 million was *not* a sunk cost mistake.

A 2014 meta-analysis summarized 45 studies, from 1976-2013, which manipulated sunk costs experimentally with adequate control, yielding 100 effect sizes[7]. Effects are measured by Cohen's d, which is the difference in means in treatment and control group, divided by the pooled standard deviation. The average effect size in the progress decisions was .44 and in utilization decisions was .58 (both highly significantly above zero).

Their sample of studies was large enough to permit analysis of variables that modulate the strength of the sunk cost effect. One variable that does *not* appear to matter is familiarity with economics. Older people are less prone to sunk cost effects in utilization decisions (and see reference (8); perhaps because of enhanced future focus[9]). This age effect is especially important because 72% of the effects came from young college student subjects.

An interesting variable with mixed effects is the length of time between when a cost was first sunk, and when a later decision must be made[10]. Time passage reduces sunk costs effects for utilization decisions but increases it (more weakly) for progress decisions. One theory rationalizes the sunk cost effect as an optimal heuristic if estimated benefits are forgotten over time. If sunk costs are proxies for those forgotten benefits, then continuing projects based on their sunk costs could be rational[11]. However, the main prediction of this theory is that sunk cost effects should get stronger with

longer time passage (presuming forgetting increases with time passage too), which is certainly not the case for utilization decisions.

Some of the most conclusive studies have come from field experiments. A pioneering study gave unexpected discounts to people buying tickets to a series of six plays[2]. The people who got discounts went to fewer of the first three plays (the difference then disappeared for the last three plays). A similar study offered unexpected discounts to diners at an all-you-can-eat pizza restaurant[12]. The diners who paid full price ate one more slice of pizza than those who paid less. In contrast, varying prices charged for a water purification product in Zambia did not show a sunk cost effect (in the form of higher usage by those who paid more)[13].

Analyses of field data without experimental control have also been consistent with sunk cost effects. Consider NBA basketball players who are chosen higher up in the draft, because they are expected to play better (and are usually paid more). Controlling for actual performance, the higher-drafted players play more minutes even three seasons later[5,14]. Driving a car in Singapore could create sunk cost effects because licenses to drive are extremely expensive[15]. Indeed, one particularly careful analysis showed that drivers who paid more for a license drove more (an effect which grew smaller over time as their license fees were mentally 'amortized'). In penny auctions, players pay a nonrefundable fee to top the bid of a previous "leader". If enough time passes after a topping bid the auction ends. The selling company collects the nonrefundable fees and sells the good at the last price. Analysis of bids indicates that players who have sunk a lot of costs (in the form of bid fees) then bid more aggressively, as if the object's value is increasing in the amount of sunk cost[16].

There are interesting data on whether animals and children exhibit sunk cost fallacies.

An early study proposed that female digger wasps are sensitive to sunk costs, because when two females fight over a nest to which they have both brought dead katykids (for food), the one who brought more katydids wins more often[17].

However, the number of katykids are both a sunk cost and have future marginal benefit, so the digger wasp example does not clearly imply a suboptimal tendency to look back at costs that are thoroughly sunk and have no association with marginal benefit, as in ideal experimental designs[18]. However, there is other evidence that animals act as if lever-pressing conditioned stimuli that require more effort to yield reward are more valuable, which is consistent with an effect of sunk cost on valuation[19].

Finally, there is some intriguing evidence, albeit from tiny samples, that 5-6 year-olds do not exhibit a sunk cost effect in the "lost ticket" (snowstorm) paradigm described above[20,21]. Eight-to-nine and 11-12 year-olds exhibit close to adult behavior, preferring to buy another ticket if they lost their ticket but not if they lost money. Younger children typically do not make decisions about how to spend scarce resources (such as money, or taking time to cook food). The development of a "no waste" heuristic, as a device to promote careful planning of resource spending, is therefore of little use to younger children. On this presumption, the unusual *lack* of a sunk cost effect among the 5-6 year olds is consistent with the general "no waste" explanation that drives adult behavior.

## Supplementary Results

### Additional analyses using the revised sunk cost effect measure

Because some participants showed preference reversals in some trials of the control condition, we also analyzed the data using the sunk cost measure taking this into account [revised sunk cost measure (revised SC): the rates of preference reversals under sunk cost condition – those under control condition]. Revised SC was significantly correlated with the agreeableness, conscientiousness, and averaged scores of these personality domains ($r = 0.51$, $p = 0.003$, $r = 0.35$, $p = 0.046$, and $r = 0.53$, $p = 0.002$, respectively, see Supplementary Table S6). Activation in the left insula also mediated the relationship of these personality traits (agreeableness, conscientiousness, and averaged scores of these personality domains) with the revised SC (Supplementary Fig. S2).

### Additional analyses concerning activation associated with decision-making under sunk costs

In the current task, the ticket prices were systematically higher in the sunk cost condition compared with the control condition because the ticket price for the non-preferred destination was presented as 50% higher than the ticket price from the preferred one in the sunk cost condition. Therefore, we performed additional analyses reducing the confounding effect of the ticket prices. Firstly, we separated the sunk cost and control trials according to their ticket prices as follows [sunk cost trials (A), sunk cost trials (B), control trials (C), control trials (D)].

*Sunk cost trials (A)*: sunk cost trials with costs of the preferred (chosen in the preference phase) options of ¥30,000, ¥40,000, ¥50,000, or ¥80,000 [costs of the non-preferred (not chosen in the preference phase) options were ¥45,000, ¥60,000, ¥75,000, or ¥120,000, respectively].

*Sunk cost trials (B)*: sunk cost trials with costs of the preferred (chosen in the preference phase) options of ¥100,000 [costs of the non-preferred (not chosen in the preference phase) options were ¥150,000].

*Control trials (C)*: control trials with costs of the options of ¥40,000, ¥50,000, ¥80,000, or ¥100,000 (both options were identical in the control trials).

*Control trials (D)*: control trials with costs of the options of ¥30,000.

The mean costs of the non-preferred options and the sum of both (preferred and non-preferred) tickets in the "sunk cost trials (A)" were ¥70,000 and ¥116,667, respectively. The mean costs of the non-preferred options and the sum of both tickets in the "control trials (C)" were ¥75,000 and ¥150,000, respectively. Therefore, (1) the mean costs of the non-preferred options and (2) the mean costs of the sum of both tickets in the "sunk cost trials (A)" were nearly equal to those of the "control trials (C)" [those in "the sunk cost trials (A)" were even slightly lower than those in the "control trials (C)"].

Then, we analyzed the data using the following GLM, which included

1. sunk cost trials (A)
2. sunk cost trials (B)
3. control trials (C)
4. control trials (D)
5. preference phase

The GLM also included motion parameters as regressors of no interest. We compared the brain activation during decision-making in the "sunk cost trials (A)" and the "control trials (C)". We also found that our identified regions during sunk cost decision-making (e.g., insula, IFG and ACC) were robustly activated [the statistical threshold was defined at a cluster-level $q < 0.05$ after correcting for multiple comparisons using FDR (at voxel-level uncorrected $p < 0.001$), Supplementary Table 7].

**Supplementary Methods**

**fMRI data acquisition and pre-processing**

All participants underwent MRI scans in a 3-T whole-body scanner equipped with an eight-channel phased array head coil (Trio; Siemens, Erlangen, Germany). Functional images were obtained in a T2*-weighted gradient-echo echo-planar imaging (EPI) sequence. The image-acquisition parameters were as follows: repetition time (TR) = 2.4 s; echo time (TE) = 30 ms; flip angle (FA) = 90°; field of view (FOV) = 192 × 192 mm; matrix = 64 × 64; 38 interleaved axial slices with 3-mm thickness without gaps (3-mm cubic voxels). The first two volumes were not saved to allow for signal stabilization, and the subsequent 349 volumes were acquired. Each participant lay supine on a scanner bed with a button-response device held in the right hand. The participant's vision was corrected, and foam pads were used to reduce head motion. Participants viewed visual stimuli that were back-projected onto a screen through a built-in mirror.

Image processing was performed using SPM8 (Wellcome Trust Center for Neuroimaging, London, UK) in MATLAB (MathWorks, Natick, MA, USA). Functional images were corrected for differences in slice-acquisition timing and were then spatially realigned to correct for head motion. The realigned images were spatially normalized to fit the EPI template supplied in SPM8. The functional images were resampled into 2 mm × 2 mm × 2 mm voxels during the normalization process. Finally, all EPI images were smoothed using a Gaussian kernel with a full width at half maximum of 8 mm in the x, y, and z-axes.

## Supplementary References


1. Staw, B.M. Knee-deep in the big muddy: A study of escalating commitment to a chosen course of action. *Organ. Behav. Hum. Decis. Process.* **16***,* 27-44 (1976).
2. Thaler, R. Toward a positive theory of consumer choice. *J. Econ. Behav. Organ.* **1**, 39-60 (1980).
3. Arkes, H.R. & Blumer, C. The psychology of sunk cost. *Organ. Behav. Hum. Decis. Process.* **35**, 124-140 (1985).
4. Friedman, D., Pommerenke, K., Lukose, R., Milam, G., & Huberman, B.A. Searching for the sunk cost fallacy. *Exp. Econ.* **10**, 79-104 (2007).
5. Camerer, C.F., & Weber, R.A. The econometrics and behavioral economics of escalation of commitment: A re-examination of Staw and Hoang's NBA data. *J. Econ. Behav. Organ.* **39**, 59-82 (1999).
6. McAfee, R.P., Mialon, H.M., & Mialon, S.H. Do sunk costs matter? *Econ. Inq.* **48**, 323-336 (2010).
7. Roth, S., Robbert, T. & Straus, L. On the sunk-cost effect in economic decision-making: a meta-analytic review. *Business Research*, 1-40, (2014).
8. Strough, J., Mehta, C.M., McFall, J.P., & Schuller, K.L. Are older adults less subject to the sunk-cost fallacy than younger adults? *Psychol. Sci.* **19**, 650-652 (2008).
9. Strough, J., Schlosnagle, L., Karns, T., Lemaster, P., & Pichayayothin, N. No time to waste: Restricting life‐span temporal horizons decreases the sunk‐cost fallacy. *J. Behav. Decis. Making.* **27**, 78-94 (2014).
10. Gourville, J.T., & Soman, D. Payment depreciation: The behavioral effects of

temporally separating payments from consumption. *J. Consum. Res.* **25**, 160-174 (1998).

11. Baliga, S., & Ely, J.C. Mnemonomics: the sunk cost fallacy as a memory kludge. *American Economic Journal: Microeconomics* **3**, 35-67 (2011).

12. Just, D.R. & Wansink, B. The flat-rate pricing paradox: conflicting effects of "all-you-can-eat" buffet pricing. *Rev. Econ. Stat.* **93**, 193-200 (2011).

13. Ashraf, N., Berry, J., & Shapiro, J.M. Can higher prices stimulate product use? Evidence from a field experiment in Zambia. *Am. Econ. Rev.* **100**, 2383-2413 (2010).

14. Staw, B.M., & Hoang, H. Sunk costs in the NBA: Why draft order affects playing time and survival in professional basketball. *Adm. Sci. Q.* **40**, 474-494 (1995).

15. Lew, Y.D. & Leong, W.Y. Managing congestion in Singapore—a behavioural economics perspective. *Journeys (Land Transport Authority, Singapore),* **May**, 15-22 (2009).

16. Augenblick, N. The sunk-cost fallacy in penny auctions. *Rev. Econ. Stud.* **83**, 58-86 (2016).

17. Dawkins, R., & Brockmann, H.J. Do digger wasps commit the Concorde fallacy? *Anim. Behav.* **28**, 892-896 (1980).

18. Arkes, H.R., & Ayton, P. The sunk cost and Concorde effects: Are humans less rational than lower animals? *Psychol. Bull.* **125**, 591 (1999).

19. Pattison, K.F., Zentall, T.R., & Watanabe, S. Sunk cost: Pigeons (Columba livia), too, show bias to complete a task rather than shift to another. *J. Comp. Psychol.* **126**, 1 (2012).

20. Krouse, H.J. Use of decision frames by elementary school children. *Percept. Mot. Skills.* **63**, 1107-1112 (1986).

21. Webley, P., & Plaisier, Z. Mental accounting in childhood. *Citizenship, Social and Economics Education* **3**, 55-64 (1998).
